# An Optimal Artificial Intelligence System for Real-Time Endoscopic Prediction of Invasion Depth in Early Gastric Cancer

**DOI:** 10.3390/cancers14236000

**Published:** 2022-12-05

**Authors:** Jie-Hyun Kim, Sang-Il Oh, So-Young Han, Ji-Soo Keum, Kyung-Nam Kim, Jae-Young Chun, Young-Hoon Youn, Hyojin Park

**Affiliations:** 1 Department of Internal Medicine, Gangnam Severance Hospital, Yonsei University College of Medicine, Seoul 06273, Republic of Korea; 2 Waycen Inc., Seoul 03722, Republic of Korea

**Keywords:** gastric cancer, artificial intelligence, convolutional neural networks, video, endoscopy

## Abstract

**Simple Summary:**

We previously constructed a VGG-16-based artificial intelligence (AI) model (image classifier [IC]) to predict the invasion depth in early gastric cancer (EGC) using static images. However, images cannot capture the spatio-temporal information available during real-time endoscopy. Thus, we constructed a video classifier [VC] using videos by attaching sequential layers to the last convolutional layer of the IC. We computed the standard deviation (SD) of output probabilities for a video clip and the sensitivities in the manner of frame units to observe consistency. The sensitivity, specificity, and accuracy of the IC for video clips were 33.6%, 85.5%, and 56.6%, respectively. The VC performed better analysis of the videos (sensitivity 82.3%, specificity 85.8%, and accuracy 83.7%, respectively). Furthermore, the mean SD was lower for the VC than the IC. The AI model developed utilizing videos can predict invasion depth in EGC more precisely and consistently than image-trained models, and is more appropriate for real-world situations.

**Abstract:**

We previously constructed a VGG-16 based artificial intelligence (AI) model (image classifier [IC]) to predict the invasion depth in early gastric cancer (EGC) using endoscopic static images. However, images cannot capture the spatio-temporal information available during real-time endoscopy—the AI trained on static images could not estimate invasion depth accurately and reliably. Thus, we constructed a video classifier [VC] using videos for real-time depth prediction in EGC. We built a VC by attaching sequential layers to the last convolutional layer of IC *v*2, using video clips. We computed the standard deviation (SD) of output probabilities for a video clip and the sensitivities in the manner of frame units to observe consistency. The sensitivity, specificity, and accuracy of IC *v*2 for static images were 82.5%, 82.9%, and 82.7%, respectively. However, for video clips, the sensitivity, specificity, and accuracy of IC *v*2 were 33.6%, 85.5%, and 56.6%, respectively. The VC performed better analysis of the videos, with a sensitivity of 82.3%, a specificity of 85.8%, and an accuracy of 83.7%. Furthermore, the mean SD was lower for the VC than IC *v*2 (0.096 vs. 0.289). The AI model developed utilizing videos can predict invasion depth in EGC more precisely and consistently than image-trained models, and is more appropriate for real-world situations.

## 1. Introduction

Several studies have reported the usefulness of artificial intelligence (AI) in diagnostic imaging, including endoscopic imaging, to detect or diagnose neoplastic lesions. However, the role of artificial intelligence in endoscopy is distinct from that of other imaging modalities, such as computed tomography or magnetic resonance imaging [1]. In other medical devices, AI can detect and diagnose lesions from saved images after the procedure has been performed. In contrast, in endoscopy, simultaneous and continuous support of AI is essential while the patient is undergoing the procedure to enable reliable evaluation when continuous frames are being sequentially captured.

All previous studies have used endoscopic static images to train AI models for detection and diagnosis of early gastric cancer (EGC) [2]. Previously, we also developed a convolutional neural network (CNN) model to detect EGC and predict the depth of invasion using static images [3]. In our previous model, the sensitivity, specificity, and positive predictive value (PPV) for EGC detection were 91.0%, 97.6%, and 97.5%, respectively. The sensitivity, specificity, and PPV of depth prediction were 79.2%, 77.8%, and 79.3%, respectively. The performance of depth prediction will be further improved.

The differentiation between mucosal and submucosal invasion was used to predict the tumor invasion depth in EGC. It is essential to make a diagnosis from observing subtle differences between two invasion depths, compared with the detection of EGC from normal mucosa. In real-world situations, endoscopists can predict invasion depth more precisely and reliably when observing subtle changes in the EGC lesion during an endoscopic procedure rather than still images of the lesion. Furthermore, since fine-grained visual features which come from spatio-temporal information were not considered, CNN trained on static images cannot reliably estimate tumor invasion depth in EGC. The structure of a neural network is inspired by the human brain; hence, training a CNN model in the same way as the human brain is more effective. Therefore, we developed an AI model to estimate the depth of tumor invasion in EGC using endoscopic videos. This allowed us to investigate and prove training an AI using endoscopic videos was more effective for predicting the tumor invasion depth in EGC as compared to endoscopic static images.

We trained our previous CNN model using more static images to improve the depth prediction performance. In addition, we developed the AI model using endoscopic videos and compared the performances of the two AI models using endoscopic videos.

## 2. Materials and Methods

### 2.1. Study Design and Data Preparation

**Figure 1:**
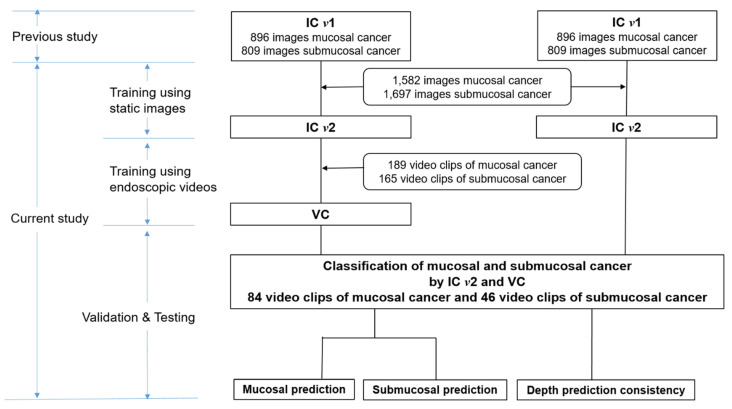
Flowchart of the study design. IC, image classifier; VC, video classifier.

The study design is summarized in Figure 1. We constructed the CNN model, which was termed as image classifier (IC) *v*2, by adding newer endoscopic static images (1582 images of mucosal cancer and 1697 images of submucosal cancer) of the training image dataset to IC *v*1, which was developed in a previous study [3]. We used independent static images of EGC (1060 images of mucosal cancer and 1060 images of submucosal cancer) as the testing set to evaluate the improvement in IC *v*2 for depth prediction.

In addition, we built a CNN model called the video classifier (VC) to analyze the data from endoscopic white-light videos. The used video data were captured for 20 s at 30 frames per second (FPS) because a lesion has to be intactly placed on whole frames composing a video. A single EGC lesion was centered on each video. To use the video data before training the model, we organized video clips by setting up the exclusion criteria. Because extreme scene changes in a video can disrupt sequential continuity, we split the video into two video clips when a discontinuity was recognized. To avoid presenting endoscopy recordings to the VC that involved occlusion of the lesion, videos containing medical procedures and/or bleeding scenes were omitted. Finally, a consecutive frame section in which an EGC lesion was detected by the EGC detector developed in previous study [3] for more than 5 s was clipped from 600 frames (20 s × 30 FPS). In total, 354 video clips of EGC (189 video clips of mucosal cancer and 165 video clips of submucosal cancer) were used to train the model. Independent video clips of EGC (40 video clips of mucosal cancer and 23 video clips of submucosal cancer) were used as the validation set. After training, the model was evaluated using the test set (44 video clips of mucosal cancer and 23 video clips of submucosal cancer).

To reflect the diversity of the video data and thus minimize the overfitting, we employed some image data augmentation methods to the training video dataset. Random flipping, random rotation, random gaussian blur, random motion blur, and sequence reverses were used to generate augmented video clips. In general, there are many types of augmentation techniques including elastic transformation and random cropping. However, the continuity which is the basic nature of video data, makes it hard to apply image augmentation methods to videos because the augmentation process may disrupt sequential information.

All endoscopic static images and videos in the current study were retrospectively consecutively collected at the Gangnam Severance Hospital, Yonsei University College of Medicine, Seoul, Korea. Baseline clinicopathological characteristics of EGC for IC *v*2 and the VC are shown in Table 1. Static images and endoscopic videos were obtained using standard endoscopes (GIF-Q260J, GIF-H260, and GIF-H290; Olympus Medical Systems Co. Ltd., Tokyo, Japan). This study was approved by the institutional review board of Gangnam Severance Hospital (no. 3-2021-0341).

### 2.2. Image Classifiers v1 and v2

A 16-layer network of the Visual Geometry Group (VGG) was employed as the backbone architecture for both IC *v*1 and IC *v*2. IC *v*1 was designed to mimic the architecture of VGG-16 [3], and some layers were replaced on IC *v*2. The differences between the two classifiers are shown in Figure S1. To reduce the overfitting possibility caused by unbalances between the number of parameters and the number of training data, we replaced the flattening operation used for traditional CNN feed flows to a global average pooling (GAP) layer on IC *v*2. The GAP layer measures the average values of the output from the last convolutional layer in a two-dimensional manner. Although the number of the training parameters was dynamically decreased by using the GAP layer, there was a known result that the performance losses were insignificant [4]. After flattening the output of the final convolutional layer, the two intermediate fully connected dense layers were skipped from the architecture. The IC parameters were optimized using the lesion-based training procedure proposed in our previous study^3^ with an adaptive moment estimation (ADAM) optimizer.

### 2.3. The Video Classifier

**Figure 2:**
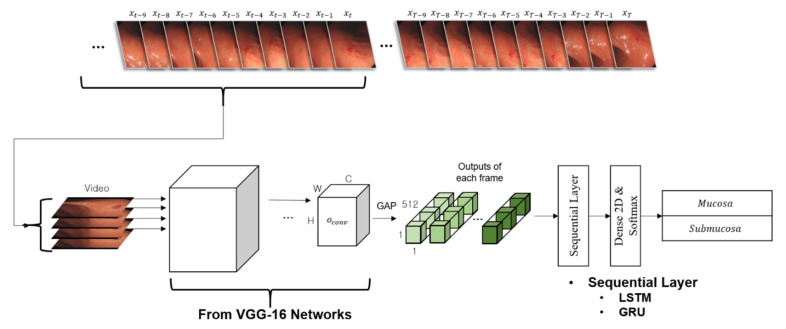
The network of the video classifier. The preliminary layers as convolutional layers have been formed by the all convolutional layers of visual geometry group (VGG)-16 networks. Sequential frames from a video clip were independently fed into the convolutional layers. All outputs from the last convolutional layer were stacked to be used as an input feature for a sequential layer. In this study, we have applied a long short-term memory (LSTM) layer and a gated recurrent unit (GRU) layer for each sequential-CNN type. Finally, two probability scores ranging from 0 to 1 for invasion depth of the input sequence were predicted through the subsequent fully connected dense layer(s) and a softmax layer.

To prove the effectiveness of utilizing endoscopic videos for training the deep learning models, we constructed and evaluated two representative sequential models. Figure 2 presents an overview of the models. The preliminary convolutional layers were formed using all the convolutional layers of the VGG-16 networks. Sequential frames from the video clip were independently fed into the convolutional layers. All outputs from the last convolutional layer were flattened through the GAP layer, and concatenated for use as input features for a sequential layer. In this study, we applied a long short-term memory (LSTM) layer and gated recurrent unit (GRU) layer for each sequential model. The LSTM layer introducing a short-term state and a long-term state to the cell was proposed to overcome the long-term dependency problem of the traditional recurrent neural network (RNN), where the information is gradually faded out by an increase in timesteps [5]. The GRU layer is a streamlined version of the LSTM layer [6]. For the GRU layer, the short-term state and long-term state of the LSTM layer were integrated into a single parameter. Both layers have been widely adopted to the AI models covering sequential problems. Finally, two probability scores for the invasion depth of the input sequence, which ranged from 0 to 1, were predicted through the subsequent fully connected dense layer(s) and a softmax layer.

To train and test the deep learning models, we used TensorFlow (version 2.4) for Python (version 3.7.10) as a backend. The models were trained on dual NVIDIA GeForce RTX 3090 with 24 GB of CUDA memory for each, whereas an NVIDIA GeForce RTX 2080Ti with 11 GB of CUDA memory was used to evaluate the performance. The weights of the convolutional layers of each VC were initialized and fixed using the weights of the convolutional layers from IC *v*2. The sequential layer and fully connected dense layer(s) were trained using an ADAM optimizer with an initial learning rate of 0.0001 on the gathered video clip dataset. To measure the training losses, we applied a cross-entropy loss. In this study, the VCs used seven consecutive frames as input frame segments. The performance differences by changing the number of input frames are shown in Figure S2.

### 2.4. Outcome Measures

A single inference procedure of ICs outputs the probabilities of an input frame, whereas the output of the VCs is the probability of the last frame among consecutive input frames. Therefore, the output of the VCs represents the predicted invasion depth of the seventh frame in this study.

To evaluate the performance of invasion depth prediction, we derived three types of final decisions from the outputs of each classifier as follows: (1) frame-by-frame prediction, (2) video clip-based prediction by averaging outputs, and (3) video clip-based prediction by voting outputs. For frame-by-frame prediction, we assumed that the invasion depth in all frames was the same as in the source video clip. To evaluate the frame-by-frame prediction, we counted the frames that were classified to have the correct invasion depth. The prediction from the video clip was deduced using the outputs of all the frames that constituted a video clip. There are two methods for determining the video clip-based prediction by the classifiers—averaging (2) and voting (3). In the averaging method, all probabilities were averaged to determine the maximum argument, whereas the voting method was used to determine the most frequent invasion depth that was estimated from among the all-frame results, which was denoted as the estimated invasion depth of EGC.

To prove that consistency increased with the video data, we also examined the probability distribution for the frame-by-frame predictions.

The class activation maps (CAM) were designed to identify the discriminative image regions which are highly contributing to the final classification result of CNNs [7]. To extract CAM, the output of the last convolutional layer was multiplied by the weight matrix of the last fully connected layer. Each component of CAM has a value ranging from 0 to 1, and the higher component value indicates the more significant impact on the final decision of the model. Using this concept, we extracted boundaries from CAM by thresholding the component values to 0.5 to visualize the activated regions.

### 2.5. Statistical Methods

The diagnostic performance of the CNN system per image and frame was measured in terms of accuracy, sensitivity, specificity, positive predictive value (PPV), negative predictive value (NPV), and area under the curve (AUC) of the receiver operating characteristic (ROC) graph. The standard deviation (SD) of the output probabilities was computed using a video clip, and the sensitivities in the manner of frame units to observe consistency.

## 3. Results

### 3.1. Diagnostic Performance of IC v1 and v2 for Static Images

Using the testing set, consisting of independent static images of EGC (1060 images of mucosal cancer and 1060 images of submucosal cancer), we evaluated the diagnostic performance of IC *v*1 and *v*2 in predicting invasion depth (Table 2). The accuracy, sensitivity, specificity, PPV, and NPV of IC *v*1 were 79.8%, 79.2%, 77.8%, 79.3%, and 77.7%, respectively. The values of IC *v*2 were 82.7%, 82.5%, 82.9%, 82.9%, and 82.6%, respectively, which showed a better performance than IC *v*1.

### 3.2. Diagnostic Performance of IC v2 and the VC for Endoscopic Videos

The diagnostic performance of IC *v*2 and the VC with GRU layers were compared in terms of frame-by-frame and video clip-based prediction when endoscopic videos (44 clips of mucosal cancer and 23 clips of submucosal cancer) were used for testing. (Table 3). The diagnostic performance of the VC with LSTM layers is described in Table S2. According to the frame-by-frame prediction (Table 3A), accuracy, sensitivity, specificity, PPV, NPV, and AUC were 56.6%, 33.6%, 85.5%, 74.4%, 50.6%, and 0.615, respectively, in IC *v*2, and 83.7%, 82.3%, 85.8%, 88.0%, 79.4%, and 0.865, respectively, in the VC with GRU layers. Video clip-based prediction was evaluated using two methods: averaging output values and the voting outputs method (Table 3B). For video clip-based prediction by averaging of output values, the accuracy, sensitivity, specificity, PPV, and NPV were 50.8%, 25.0%, 100.0%, 100.0%, and 41.1%, respectively, in IC *v*2, and 85.1%, 81.8%, 91.3%, 94.7%, and 72.4%, respectively, in the VC with GRU layers. By the voting output method, the accuracy, sensitivity, specificity, PPV, and NPV were 50.8%, 25.0%, 100.0%, 100.0%, and 41.1%, respectively, and for the VC with GRU layers, the respective values were 82.1%, 81.8%, 82.6%, 90.0%, and 70.4%.

### 3.3. Diagnostic Consistency between IC v2 and the VC for Endoscopic Videos

**Figure 3:**
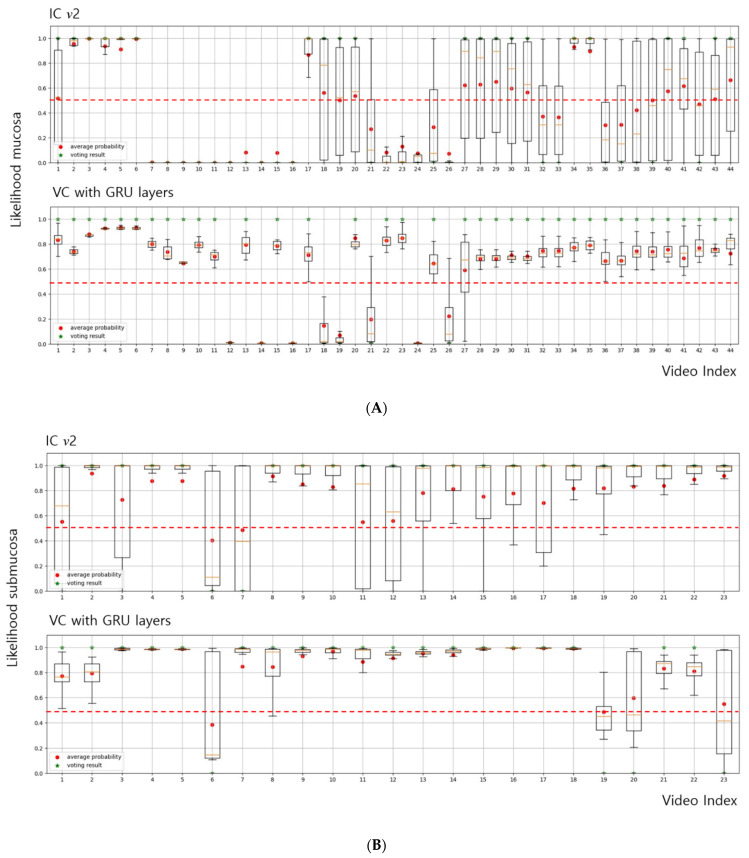
The probability distribution in frame-by-frame prediction results between IC *v*2 and the VC with GRU layers on (**A**) mucosa videos and (**B**) submucosa videos. IC, image classifier; VC, video classifier. The box plots are the probability distribution of each video for each invasion depth. The red dashed lines are a cut-off value to decide the correct prediction. In this study, we set the cut-off value to 0.5 because it has been widely used as a threshold value in a binary classification task. The red circles and green stars refer to the average probabilities and the voting results, respectively. In the many frames of IC *v*2, the likelihood of depth prediction showed both extremes between mucosa and submucosa. In contrast, the likelihood of depth prediction was more reliable in the VC (mean SD of output probabilities in the VC vs. IC *v*2, 0.096 vs. 0.289).

Figure 3 shows the probability distribution in the frame-by-frame prediction results between IC *v*2 and the VC with GRU layers. The mean SD of the output probabilities was lower for the VC-based analysis than for IC *v*2 (0.096 vs. 0.289). That is, in many frames of IC *v*2, the invasion depth prediction showed values ranging between both extremes spanning the mucosa and submucosa. In contrast, the likelihood of depth prediction was more reliable with the VC. These findings indicate that the VC has higher diagnostic consistency than the IC. Videos S1 and S2 show the differences between IC *v*2 and the VC in real-world situations.

## 4. Discussion

Many studies have reported the development of AI models to detect gastric cancer, including our previous study, which showed favorable results [2,3,8,9,10,11,12]. All studies have used endoscopic static images to train AI models, whereas few have used endoscopic videos to test the AI model. However, the frame selection was suspicious and insufficient to arrive at a real-time simultaneous diagnosis during the procedure. The most important feature of real-time endoscopy is the availability of spatio-temporal information, especially temporal information, which differs from other static imaging modalities. The 3D reconstruction of static images can provide spatial information; however, temporal information can only be provided from the sequential frames extracted from real-time video. In real-world situations, endoscopists perform endoscopic procedures and make the diagnoses simultaneously. Hence, it is necessary to train AI models using endoscopic videos to develop an optimal AI model for real-world endoscopic environments. In this study, we developed a CNN model and trained it using endoscopic videos, which resulted in a better and more consistent performance than the image-trained model.

Several CNN models were developed to predict the invasion depth in EGC [3,13,14,15,16,17]. All CNN models showed good performance in predicting the tumor invasion depth in EGC from static endoscopic images, including our previous model [3]. However, no study has evaluated the diagnostic consistency of endoscopic videos to predict invasion depth in EGC. Diagnostic consistency should be assessed to appraise diagnostic performance precisely. The evaluation of each still image or frame-selected video cannot accurately reflect real-world scenarios. In the present study, we improved the ability of our previous model (IC *v*1) to predict the invasion depth by training it with more static images. However, the performance deteriorated when testing was performed using endoscopic videos. Furthermore, the diagnostic performance was very unreliable, and values representing both extremes between the mucosa and submucosa (Figure 3, Videos S1 and S2) were depicted. If the answer frames were selected, the CNN model would have high diagnostic performance. However, real-world situations are different. Our VC model, trained using videos, could predict the tumor invasion depth more reliably and accurately than an image-trained model (IC *v*2), as observed in the unedited video (Videos S1 and S2). The role of AI during the endoscopic procedure is decision support [18,19]. If the AI can give us information about the exact invasion depth during endoscopy, the endoscopist can make a final decision to predict the invasion depth easily and rapidly. Thus, in the real-world endoscopic environment, AI must give consistent and exact information to endoscopists. Accordingly, we developed the optimal AI model to predict the invasion depth in EGC more accurately and consistently. Our AI model can give endoscopists exact and consistent information during an endoscopic procedure.

To observe the effectiveness of utilizing video data containing spatial and temporal information, we modeled two types of video classifiers using sequential layers. In this study, we attached an LSTM and GRU to the tail of the convolutional layers. The architectures of the convolutional layers and their trained weights were adopted from the pre-trained IC *v*2 for the front layers of the two video classifiers. Several sequential frames were independently fed into the convolutional layers to extract spatial features. Subsequently, the sequential layer analyzed the spatial-temporal relationship using sequentially stacked convolutional outputs as the input. Similar to our concept, the algorithms to detect anatomical landmark in the upper endoscopy have been proposed for temporal correlations [20,21,22,23,24]. CNN models can detect anatomical sites in the upper gastrointestinal tract but could not explain the temporal correlations among the frames. Thus, the algorithms have been proposed to consider temporal correlations.

Generally, the number of training datasets that are employed determines the efficiency of deep learning. That is, the performance of deep learning grows with increasing data [25,26,27,28]. Namikawa et al. constructed an advanced CNN model by adding more training datasets that consisted of benign lesions, to improve the low PPV of the original CNN model in detecting gastric cancer [29]. They could enhance the PPV from 30.6 to 92.5% [8,29]. The size of the dataset is significant for developing a CNN algorithm [30]. However, the types of datasets are also crucial for developing a CNN algorithm that reflects real-world situations. Our data suggest that an AI algorithm trained using endoscopic videos may be more appropriate for predicting the invasion depth of EGC in a real-time endoscopic environment. However, our data need to be externally validated using many video images.

## 5. Conclusions

We developed an AI model utilizing endoscopic videos which can predict the invasion depth of EGC more precisely and consistently than an image-trained model. It is more appropriate for real-world endoscopic situations.

## Figures and Tables

**Table 1:** Baseline clinicopathological characteristics of early gastric cancer for image classifier (IC) *v*2 and the video classifier (VC).

Characteristics	IC *v*2 (*N* = 714)	VC (*N* = 81)
Tumor size (mm, mean ± SD)	23.7 ± 14.4	31.0 ± 19.3
Location (*n*, %)		
Upper one-third	58 (8.2)	9 (11.1)
Middle one-third	171 (23.9)	12 (14.8)
Lower one-third	485 (67.9)	60 (74.1)
Gross type (*n*, %)		
Elevated	111 (15.5)	19 (23.4)
Flat	331 (46.4)	37 (45.7)
Depressed	272 (38.1)	25 (30.9)
* Depth of invasion (*n*, %)		
Mucosa (T1a)	426 (59.7)	50 (61.7)
Submucosa (T1b)	288 (40.3)	31 (38.3)
Japanese classification (*n*, %)		
Differentiated	419 (58.7)	45 (55.6)
Undifferentiated	295 (41.3)	36 (44.4)

* Confirmed by pathologic findings after endoscopic or surgical resection.

**Table 2:** Diagnostic performance for depth prediction of image classifier (IC) *v*1 and *v*2 for static images.

Predicting Depth	IC *v*1	IC *v*2
Accuracy (%)	79.8	82.7
Sensitivity (%)	79.2	82.5
Specificity (%)	77.8	82.9
PPV (%)	79.3	82.9
NPV (%)	77.7	82.6

PPV, positive predictive value; NPV, negative predictive value.

**Table 3:** Diagnostic performance of depth prediction of image classifier (IC) *v*2 and the video classifier (VC) with GRU layers for endoscopic videos by (A) frame-by-frame prediction (B) video clip-based prediction.

(A)				
Predicting Depth	IC *v*2		VC	
Accuracy (%)	56.6		83.7	
Sensitivity (%)	33.6		82.3	
Specificity (%)	85.5		85.8	
PPV (%)	74.4		88.0	
NPV (%)	50.6		79.4	
AUC	0.615		0.865	
**(B)**				
Predicting depth	Voting		Average	
	IC *v*2	VC	IC *v*2	VC
Accuracy (%)	50.8	82.1	50.8	85.1
Sensitivity (%)	25.0	81.8	25.0	81.8
Specificity (%)	100.0	82.6	100.0	91.3
PPV (%)	100.0	90.0	100.0	94.7
NPV (%)	41.1	70.4	41.1	72.4

(A) PPV, positive predictive value; NPV, negative predictive value; AUC, area under the curve. (B) PPV, positive predictive value; NPV, negative predictive value.

## Data Availability

The corresponding author will provide the data presented in this study upon reasonable request.

## Supplementary Materials

The following supporting information can be downloaded at: https://www.mdpi.com/article/10.3390/cancers14236000/s1, Figure S1: The architectures of the image classifiers (IC) v1 and v2. IC v1 mimicked the architecture of the visual geometry group (VGG)-16,3 while some layers had been replaced on IC v2; Figure S2: The performance differences by varying the number of input frames. Both on the perspective of performances and computations costs, taking consecutive seven frames as the input of the network was relatively suitable for experiments; Table S1: Diagnostic performance of depth prediction of video classifier (VC) with LSTM layers for endoscopic videos Video S1: The lesion was a mucosal-invasive gastric cancer located in the lower body. The green lines were extracted by thresholding the activation values to 0.5. To show real operation environments, we only drew green lines when a frame was classified as a lesion by the EGC detector introduced in our previous study.3 In the upper row of the video, the image classifier v2 predicted the depth unreliably, showing both extremes between the mucosa and submucosa. In contrast, in the lower row of the video, the video classifier predicted mucosal invasion more reliably; Video S2: The lesion was a submucosal invasive gastric cancer located at the antrum. The green lines were extracted by thresholding the activation values to 0.5. To show real operation environments, we only drew green lines when a frame was classified as a lesion by the EGC detector introduced in our previous study.^3^ In the video in the upper row, the image classifier *v*2 predicted the depth unreliably, showing both extremes between the mucosa and submucosa. In contrast, in the lower row of the video, the video classifier predicted submucosal invasion more reliably.
