# Supplementary material for: An Optimal Artificial Intelligence System for Real-Time Endoscopic Prediction of Invasion Depth in Early Gastric Cancer
Source: Cancers (Basel). 2022 Dec 5;14(23):6000. doi: 10.3390/cancers14236000 (PMC9741000; doi:10.3390/cancers14236000)
Supplement: Supplementary file 1 [file cancers-14-06000-s001.zip › Suppl Table S1.pdf]

Table S1. Diagnostic performance of depth prediction of video classifier (VC) with LSTM layers for endoscopic videos.

Predicting depth	Frame-by-frame	Voting	Average
Accuracy (%)	79.5	76.1	79.1
Sensitivity (%)	76.8	70.5	72.7
Specificity (%)	82.8	87.0	91.3
PPV (%)	84.9	91.2	94.1
NPV (%)	74.0	60.6	63.6
AUC	0.833		

PPV, positive predictive value; NPV, negative predictive value; AUC, area under the curve.
