# Supplementary material for: An Optimal Artificial Intelligence System for Real-Time Endoscopic Prediction of Invasion Depth in Early Gastric Cancer
Source: Cancers (Basel). 2022 Dec 5;14(23):6000. doi: 10.3390/cancers14236000 (PMC9741000; doi:10.3390/cancers14236000)
Supplement: Supplementary file 1 [file cancers-14-06000-s001.zip › Video Suppl.pdf]

Video S1. The lesion was a mucosal-invasive gastric cancer located in the lower body. The green lines were extracted by thresholding the activation values to 0.5. To show real operation environments, we only drew green lines when a frame was classified as a lesion by the EGC detector introduced in our previous study.[3] In the upper row of the video, the image classifier *v*2 predicted the depth unreliably, showing both extremes between the mucosa and submucosa. In contrast, in the lower row of the video, the video classifier predicted mucosal invasion more reliably.

Video S2. The lesion was a submucosal invasive gastric cancer located at the antrum. The green lines were extracted by thresholding the activation values to 0.5. To show real operation environments, we only drew green lines when a frame was classified as a lesion by the EGC detector introduced in our previous study.[3] In the video in the upper row, the image classifier *v*2 predicted the depth unreliably, showing both extremes between the mucosa and submucosa. In contrast, in the lower row of the video, the video classifier predicted submucosal invasion more reliably.
