# Supplementary figures and images for: An Optimal Artificial Intelligence System for Real-Time Endoscopic Prediction of Invasion Depth in Early Gastric Cancer
Source: Cancers (Basel). 2022 Dec 5;14(23):6000. doi: 10.3390/cancers14236000 (PMC9741000; doi:10.3390/cancers14236000)

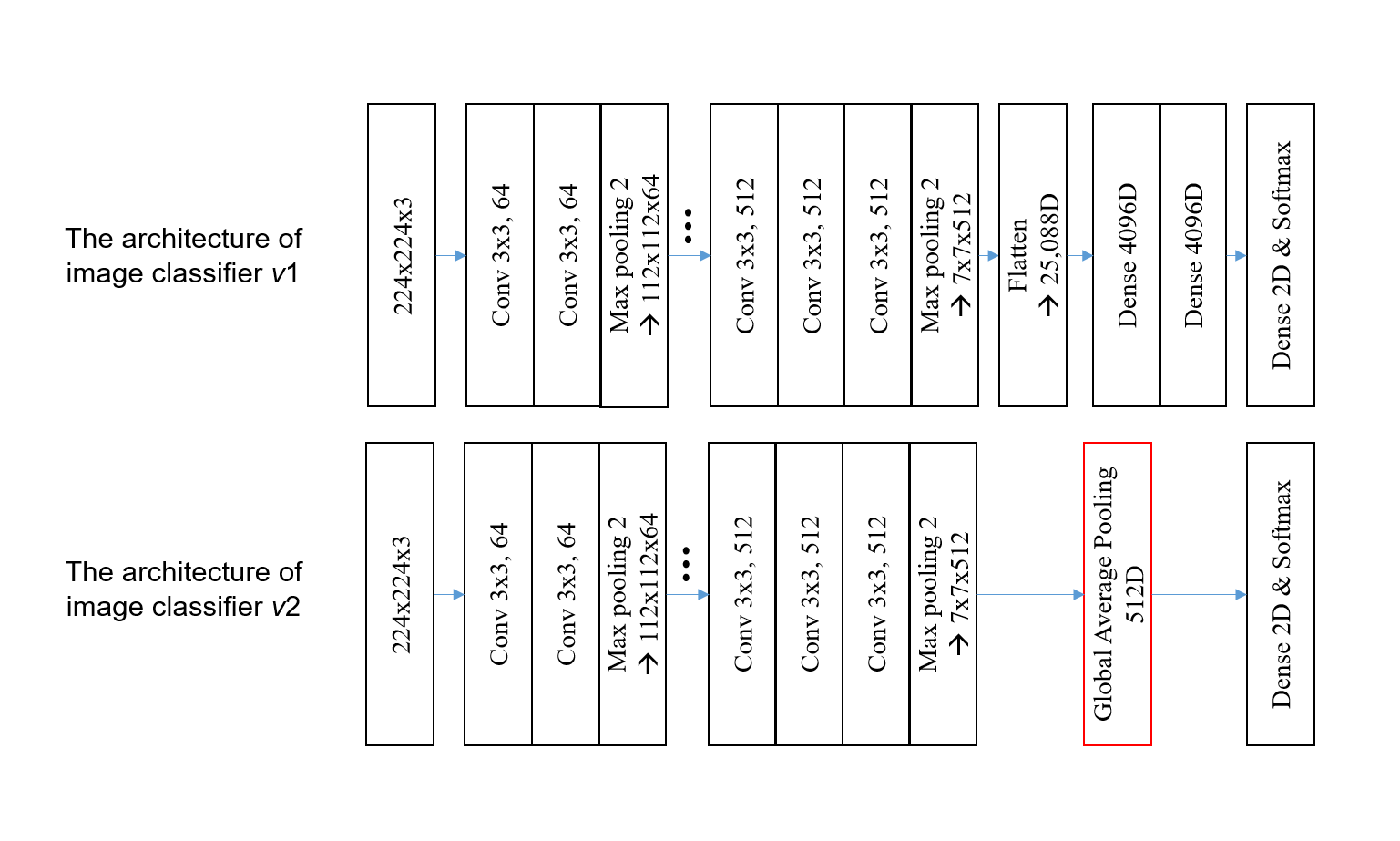

Supplement: Supplementary file 1 [file cancers-14-06000-s001.zip › Suppl Figure S1.tif]

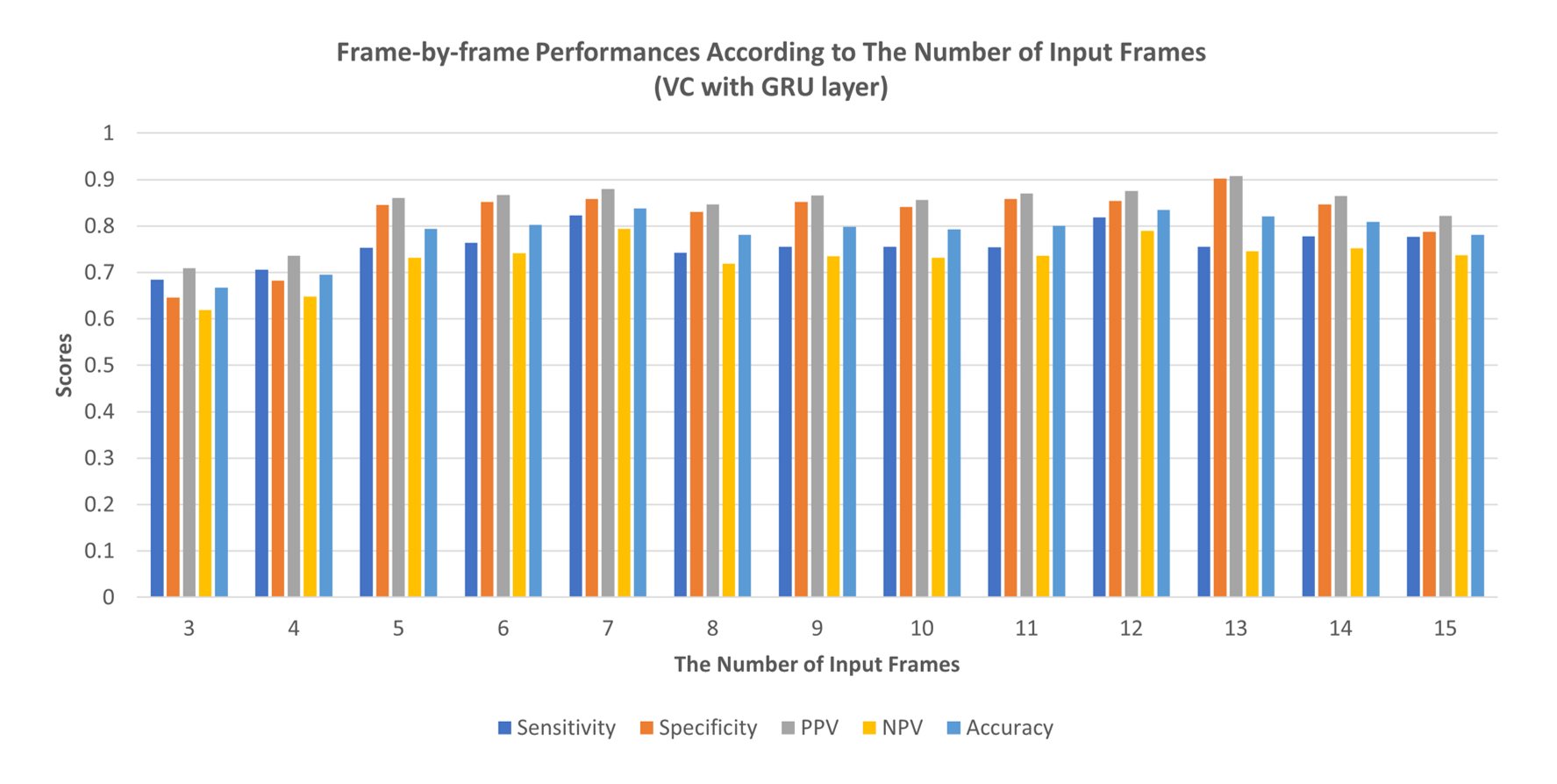

Supplement: Supplementary file 1 [file cancers-14-06000-s001.zip › Suppl Figure S2.tif]
